# Utilization of Dysphagia Services Among Older Adults Hospitalized With Pneumonia in a Large Sample of US Hospitals

**DOI:** 10.1111/jgs.70119

**Published:** 2025-09-27

**Authors:** Jordanna S. Sevitz, Meggie Griffin, Nicole Rogus-Pulia, Michael Pulia

**Affiliations:** 1 Laboratory for the Study of Upper Airway Dysfunction, Department of Biobehavioral Sciences, Teachers College, Columbia University, New York, New York, USA; 2 Department of Medicine, University of Wisconsin-Madison School of Medicine and Public Health (SMPH), Madison, Wisconsin, USA; 3 BerbeeWalsh Department of Emergency Medicine, University of Wisconsin-Madison School of Medicine and Public Health, Madison, Wisconsin, USA; 4 Department of Industrial and Systems Engineering, University of Wisconsin-Madison College of Engineering, Madison, Wisconsin, USA

**Keywords:** aspiration, dysphagia, pneumonia

## Abstract

**Background:**

Pneumonia is a major cause of morbidity and mortality among older adults, with increased risk for individuals with neurodegenerative conditions. Although dysphagia is a significant risk factor for pneumonia pathogenesis, there is a paucity of data on dysphagia management in patients hospitalized with pneumonia. Therefore, we examined dysphagia service utilization rates and associated clinical factors for older adults hospitalized with pneumonia.

**Methods:**

We identified community-acquired and aspiration pneumonia (CAP and Asp-PNA) general care hospitalizations among older adults (age ≥ 60) between April 2022 and December 2023 using the Premier Healthcare Database. Factors that influenced utilization of three primary dysphagia services—clinical evaluations, instrumental evaluations (e.g., videofluoroscopy), and therapy—were examined in three generalized linear mixed models with a random effect for hospital and adjusted for patient demographics and hospital characteristics.

**Results:**

Our sample included 195,782 older adults (51.7% female; 19.3% ND; 15.7% Asp-PNA) across 943 hospitals, and 23.6% received a clinical evaluation, 6.8% an instrumental evaluation, and 11.2% therapy. Generalized linear mixed models of clinical evaluations, instrumental evaluations, and therapy revealed significant associations of Asp-PNA, neurodegenerative disease (ND), and their interaction. The association between Asp-PNA and dysphagia services was greater in the non-ND group (clinical: OR = 9.57; instrumental: OR = 9.67; therapy: OR = 8.66) and attenuated in the ND group (clinical: OR = 5.49; instrumental: OR = 4.66; therapy: OR = 4.20). Similarly, the association between ND and dysphagia services was greater for those with CAP (clinical: OR = 2.85; instrumental: OR = 2.03; therapy: OR = 3.11) and dampened for Asp-PNA (clinical: OR = 1.64; instrumental: OR = 0.98; therapy: OR = 1.50).

**Conclusions:**

Dysphagia services were provided to less than one quarter of older adults with pneumonia in our large U.S. cohort, although rates were higher among patients with Asp-PNA and neurodegenerative disease. Future research should focus on using dysphagia screening tools to promote appropriate referrals to dysphagia services for pneumonia patients.

## 1 | Introduction

Pneumonia is a major cause of morbidity and mortality among older adults, accounting for over 1.5 million unique hospital admissions annually and yielding an annual healthcare cost of $7.7 billion in the United States (U.S.) [1, 2]. Pneumonia hospitalizations result in over 100,000 deaths per annum in the U.S. [3], with reports suggesting that in-hospital mortality from pneumonia ranges from 6.5%–10.6% [1, 3]. Longitudinal data suggest substantially higher mortality over time—with one analysis identifying that one third of patients with pneumonia died within 1 year of initial hospitalization [3]. Hospital admissions for pneumonia dramatically increase with advancing age [1, 4, 5], with particularly high incidence among older adults with neurodegenerative disease (e.g., Parkinson’s disease (PD), Alzheimer’s and Related Dementias (ADRD), etc.) [6]. In fact, pneumonia is the leading cause of death in neurodegenerative populations [7–9].

Pneumonia is typically classified into several types, reflecting differences in pathogenesis and means of contraction. These commonly include community-acquired pneumonia (CAP)—which encompasses all pneumonias acquired outside the hospital, and aspiration pneumonia (Asp-PNA)—which specifically refers to pneumonia contracted from foreign materials, such as food, liquid, or secretions, entering the lungs and resulting in bacterial colonization [10]. Although this diagnostic differentiation between general CAP and more specific Asp-PNA exists, the conceptual distinction is less clear—with considerable ambiguity and overlap between diagnoses. In fact, oropharyngeal aspiration is the most common pathway for a microbial agent to reach the lung alveoli for all types of pneumonia [3, 11]. Moreover, there are no gold-standard diagnostic criteria to distinguish Asp-PNA from CAP, and these diagnostic codes are often used inconsistently [12, 13]. Recent efforts to define this in the literature have not come to consensus and have, in fact, highlighted the lack of standardization in diagnosing CAP versus Asp-PNA [14, 15]. Provider- and institution-level differences in diagnostic approach and coding practices may determine whether a pneumonia patient receives a more general diagnosis of CAP or a more specific diagnosis of Asp-PNA. Regardless of diagnosis, studies have shown that pneumonia patients who exhibit aspiration and/or other swallowing deficits have worse healthcare outcomes, higher rates of re-hospitalization, and higher rates of mortality [16–19]. Therefore, attention to aspiration that underlies pneumonia pathogenesis is a critical aspect of clinical care for all pneumonia patients. Yet, little is known about current care patterns for evaluating and managing aspiration in hospitalized individuals with pneumonia and how this may differ when patients are diagnosed with a general CAP diagnosis versus a specific Asp-PNA diagnosis.

Speech language pathologists (SLPs) are healthcare professionals trained to determine the presence and severity of oropharyngeal aspiration and, more generally, evaluate and treat dysphagia (swallowing dysfunction). To our knowledge, three studies have examined rates of SLP service provision for pneumonia patients [5, 20, 21]. These studies have documented the provision of SLP services to 23% of older adults hospitalized with CAP [21] and to between 50% and 60% of older adults hospitalized with Asp-PNA [5, 20, 21]. This prior work has evaluated service provision in relatively small cohorts (i.e., several hundred patients) at a single institution only and lacks detail regarding the patient populations and types of dysphagia services provided.

SLPs provide three main types of dysphagia services—clinical evaluations, instrumental evaluations (e.g., videofluoroscopy), and therapy; and when dysphagia is identified, the optimal care pathway includes provision of all service types. For example, dysphagia suspected on clinical evaluation (step 1) would trigger an instrumental evaluation (step 2) to confirm safety deficits that are hypothesized based on visible signs and symptoms and identify specific pathophysiologic impairments, which would guide therapy (step 3). When dysphagia rehabilitation is initiated early, pneumonia patients experience better outcomes, including shorter hospital stays, faster return to oral diet, and a lower mortality rate [20, 22, 23]. Therefore, the present study aimed to more specifically characterize current dysphagia service delivery for older adults admitted to the hospital with pneumonia across the U.S. We retrospectively examined utilization of these three types of dysphagia services—(1) clinical evaluation, (2) instrumental evaluation, and (3) dysphagia therapy in a large national cohort of older adults who were hospitalized with community onset pneumonia, either diagnosed generally as CAP or specifically as Asp-PNA. We hypothesized that all three types of dysphagia services would be higher among those who had a neurodegenerative (ND) (vs. non-ND) diagnosis and among those with Asp-PNA (vs. CAP). We also hypothesized that service provision would be lower in rural (vs. urban) and community (vs. academic) hospitals.

## 2 | Methods

### 2.1 | Eligibility Criteria

We used the Premier Healthcare Database (PHD; Premier Inc.) to analyze the utilization of dysphagia services for hospitalized older adults with and without neurodegenerative disease diagnosed with Asp-PNA and CAP. The PHD contains deidentified data for approximately 25% of annual U.S. inpatient admissions compiled from a geographically and structurally diverse group of acute care hospitals [1]. More details on the PHD can be found in the most recent 2025 White Paper [24]. The local Institutional Review Board (IRB) determined that because all data was fully deidentified, this study was not human subjects research. We included general care admissions from April 2022 to December 2023 for patients aged 60 and above with community onset pneumonia operationalized to include all individuals with a general diagnosis of CAP and/or a specific diagnosis of Asp-PNA, which we defined as meeting the following criteria: (1) ICD-10 diagnosis code for Asp-PNA or CAP (Table S1); (2) chest imaging ordered during the emergency department visit or first day of admission; and (3) antibiotic treatment targeting pneumonia (Table S2) during the emergency department visit or first day of admission. Since Asp-PNA is the more specific diagnosis, in instances where both CAP and Asp-PNA were both coded, the patient was included in the Asp-PNA group. We excluded elective admissions, patients that received care in intermediate care (IMC; step down level of care between ICU and general ward), and intensive care units (ICU), as these patients often have other indications for dysphagia services related to being critically ill (e.g., post-extubation). We also excluded patients diagnosed with acute stroke (ICD-10 codes I60, I61, and I63) [25] given that it is standard practice to assess and treat dysphagia as part of acute stroke treatment.

### 2.2 | Study Measures and Outcomes

We considered three aspects of dysphagia service provision—(1) clinical evaluations—which refers to any bedside swallowing evaluation completed by an SLP, (2) instrumental evaluations— which refers to imaging such as a Videofluoroscopic Swallow Study (VFSS) or a Flexible Endoscopic Evaluation of Swallowing (FEES), and (3) therapy—which encompasses any dysphagia therapy provided by an SLP. Provision of each type of dysphagia service was defined according to administrative billing codes (Table S3), and we created binary (yes/no) variables for whether each service was provided during admission. To understand patterns of dysphagia service provision, we first completed a descriptive analysis, whereby we examined service provision for patients with CAP and Asp-PNA by neurodegenerative disease status (Table S4). We described patient demographics, including age, sex, race, ethnicity, primary insurance payor, and hospital characteristics including bed size, rurality, and type (academic vs. community). Because of our large sample size, we used absolute standardized differences (ASDs), the absolute difference in means divided by the pooled standard deviation, to compare characteristics of the groups. We considered an ASD greater than 10% to demonstrate meaningful differences between the groups [26–28].

### 2.3 | Statistical Analysis

**FIGURE 1 |:**
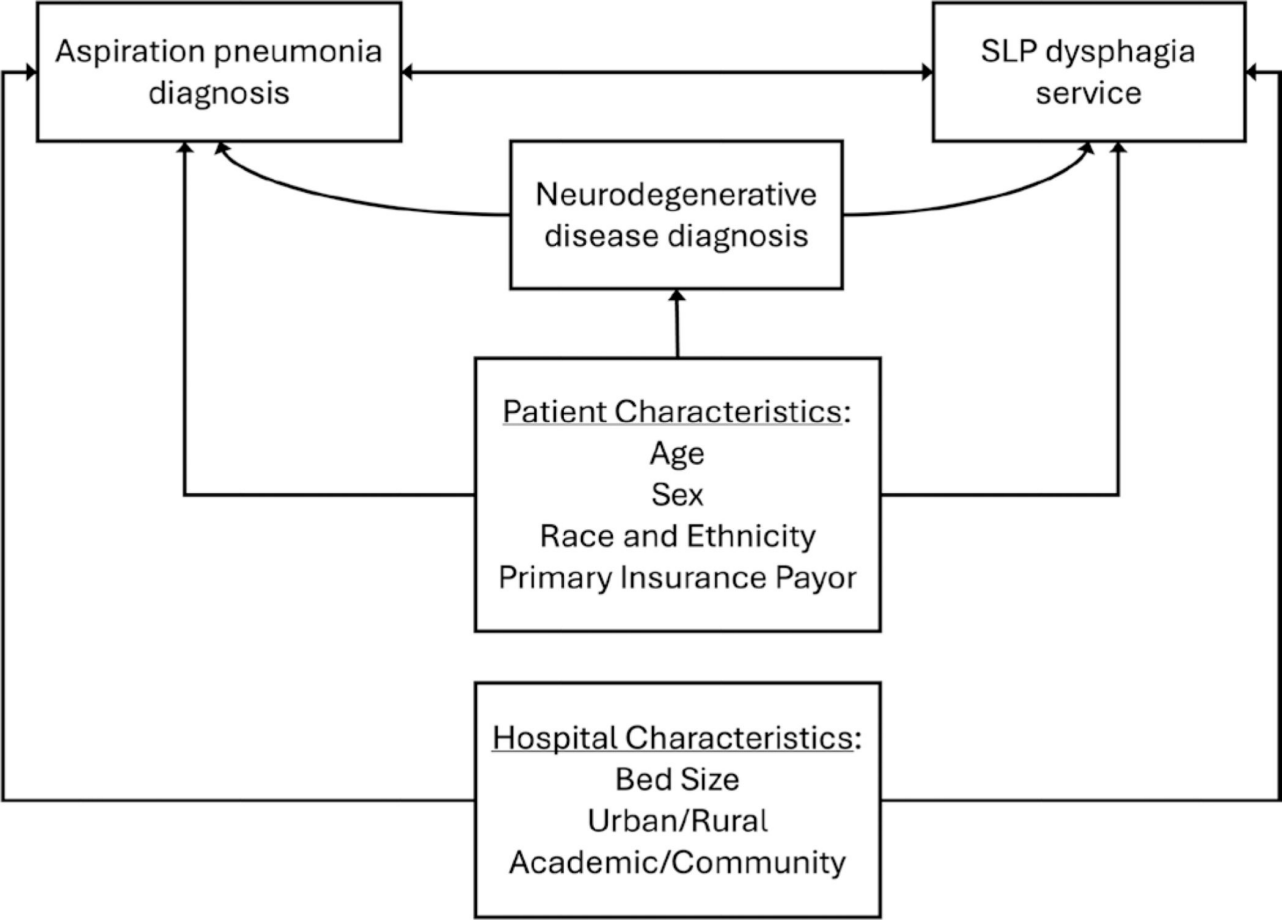
This conceptual model illustrates the hypothesized relationships between the selected variables of interest that were included in the models. Arrows indicate hypothesized unidirectional or bidirectional relationships.

To investigate factors that influenced dysphagia service utilization, we built three generalized linear mixed models (GLMM), one for each binary utilization outcome: clinical evaluation, instrumental evaluation, and therapy. The models were built based on a conceptual model (Figure 1) to illustrate the available variables we hypothesized would be associated with dysphagia service utilization and the direction of their relationships. In all models, we included indicators of neurodegenerative disease (ND vs. non-ND) and pneumonia diagnosis (Asp-PNA vs. CAP), as well as their interaction, to compare utilization between these groups. We also adjusted for patient demographics (age, sex, race and ethnicity, primary insurance payor) and hospital characteristics (bed size, rurality, type) and included a random effect for hospital to account for variation in dysphagia service utilization across sites. Model inclusion was not conditional; all participants were included in each model. Unadjusted models were also run for each predictor and outcome. Data analysis was completed in R (v.4.4.2), and the package lme4 was used to create the GLMMs [29, 30].

## 3 | Results

### 3.1 | Patient Characteristics

**FIGURE 2 |:**
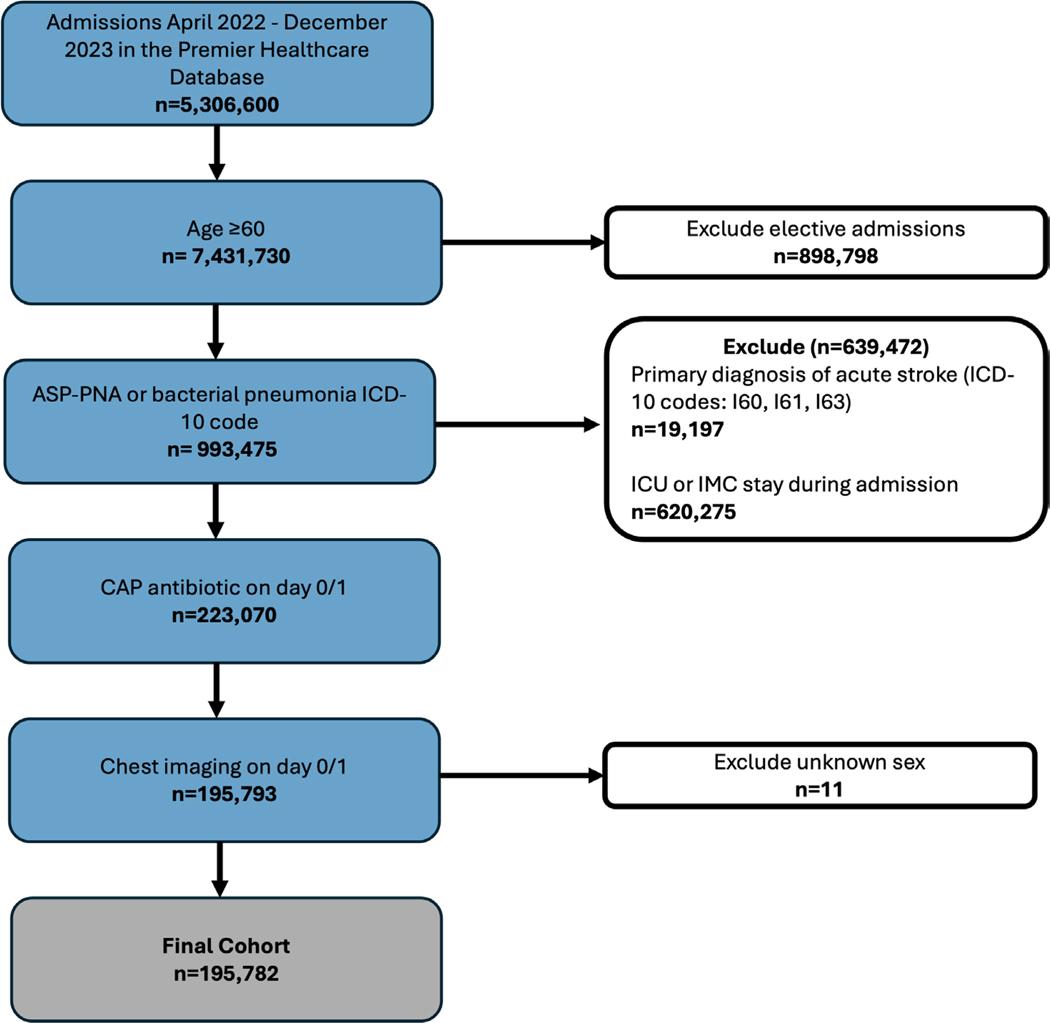
CONSORT diagram outlining the flow of participant inclusion and defining the study cohort.

**FIGURE 3 |:**
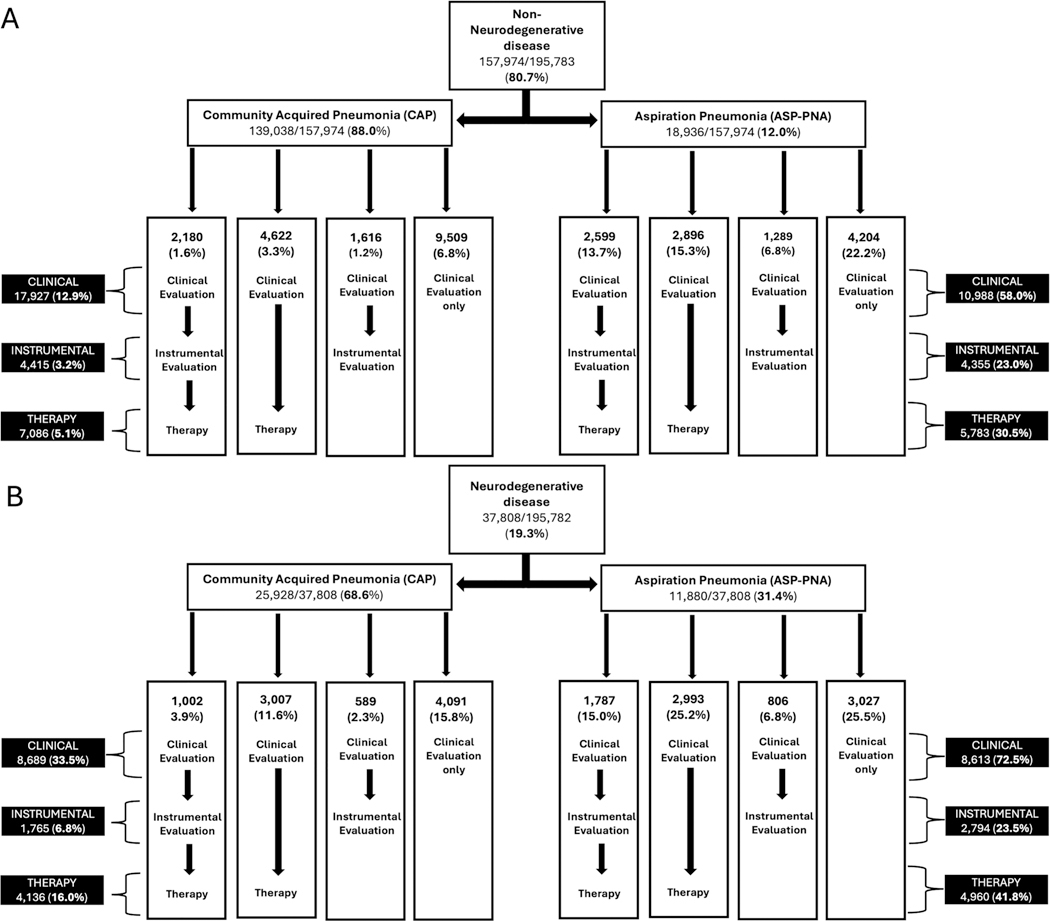
A small percentage of patients did not receive a clinical evaluation, but did receive either an instrumental evaluation, therapy, or both. Because this does not reflect standard practice, we have not included this in the pathways, but we have represented these patients in the total number of services received in the black boxes on the left and right sides of the figure. (A) 472 (0.34%) of patients in the non-ND CAP group and 250 (1.3%) of patients in the non-ND Asp-PNA group received an instrumental evaluation only. 147 (0.11%) of patients in the non-ND CAP group and 217 (1.1%) of patients in the non-ND Asp-PNA group received an instrumental evaluation and therapy.137 (0.1%) of patients in the non-ND CAP group and 71 (0.4%) of patients in the non-ND Asp-PNA group received therapy only. (B) 122 (0.47%) of patients in the ND CAP group and 95 (0.80%) of patients in the ND Asp-PNA group received an instrumental evaluation only. 52 (0.20%) of patients in the ND CAP group and 106 (0.89%) of patients in the ND Asp-PNA group received an instrumental evaluation and therapy. 75 (0.29%) of patients in the ND CAP group and 74 (0.62%) of patients in the ND Asp-PNA group received therapy only.

Our sample included 195,782 older adults across 943 hospitals (Figure 2). Mean age was 76.5 [60–89], 51.7% were female, 80.0% were White, 83.9% were insured by Medicare, 83.6% of patients were from urban hospitals, and 43% from academic hospitals. There was representation of patients across hospital size—with 34.7% from small (< 200 bed) hospitals, 28.4% from medium size hospitals (200–399 beds), and 36.9% from large hospitals (≥ 400 beds). Of all participants, 157,974 (80.7%) were in the non-ND group and 37,808 (19.3%) were in the ND group; 164,966 (84.3%) had CAP and 30,816 (15.7%) had ASP-PNA. Patients who received diagnoses of both CAP and Asp-PNA (*n* = 7753) were assigned to the Asp-PNA group. Detailed demographics for the overall sample, as well as by PNA type can be found in Table 1 and by ND type can be found in Table S5. Figure 3A,B depicts the service provision pathway for those in the non-ND (3A) and ND (3B) groups.

### 3.2 | Clinical Evaluations

Overall, 23.6% of older adults with pneumonia received a clinical (bedside) dysphagia evaluation. Higher utilization of clinical evaluations was associated with ND diagnosis (ND = 45.8%; non-ND (ref) = 18.3%) and Asp-PNA diagnosis (Asp-PNA = 63.6%; CAP (ref) = 16.1%). The interaction between Asp-PNA and ND diagnosis was significant (OR (95% CI) = 0.57 (0.54–0.61)), indicating that while Asp-PNA was associated with 9.57 times greater odds of receiving a clinical evaluation in the non-ND group (OR (95% CI) = 9.57 (9.53–9.60)), the association was dampened in the ND group (OR (95% CI) = 5.49 (5.22–5.77)). Similarly, having an ND diagnosis was associated with 2.85 times greater odds of receiving a clinical evaluation for those with CAP (OR (95% CI) = 2.85 (2.76–2.95)); however, the association between ND diagnosis and receiving a clinical evaluation was dampened among those with Asp-PNA (OR (95% CI) = 1.64 (1.55–1.72)). Patients had higher odds of receiving clinical evaluations at hospitals in urban areas (urban = 24.7%; rural (ref) = 18.1%; OR (95% CI) = 1.57 (1.41–1.75)) and lower odds at small (< 200 bed) hospitals when compared to large (≥ 400 bed) hospitals (< 200 bed = 21.1%; ≥ 400 beds (ref) = 25.5%; OR (95% CI) = 0.84 (0.74–0.96)). Utilization was also higher among males (males = 25.1%; females (ref) = 22.2%; OR (95% CI) = 1.13 (1.11–1.16)) and Asian individuals (Asian = 32.0%; White (ref) = 23.9%; Black = 21.0%; Other = 21.5%; OR (95% CI) = 1.14 (1.05–1.24)). See Table 2 for key significant adjusted model results and Table S6 for full model results.

### 3.3 | Instrumental Evaluations

Of all pneumonia patients in the study sample, 6.8% received an instrumental swallowing evaluation. Higher utilization of instrumental evaluations was associated with ND diagnosis (ND = 12.1%; non-ND (ref) = 5.6%) and Asp-PNA diagnosis (Asp-PNA = 23.2%; CAP (ref) = 3.7%). The interaction between Asp-PNA and ND diagnosis was significant (OR (95% CI) = 0.48 (0.44–0.52)), indicating that while Asp-PNA was associated with 9.67 times greater odds of receiving an instrumental evaluation in the non-ND group (OR (95% CI) = 9.67 (9.21–10.14)), the association was dampened in the ND group (OR (95% CI) = 4.66 (4.35–4.99)). Having an ND diagnosis was associated with double the odds of receiving an instrumental evaluation for those with CAP (OR (95% CI) = 2.03 (1.91–2.16)); however, the association between ND diagnosis and receiving an instrumental evaluation was not significant among those with Asp-PNA (OR (95% CI) = 0.98 (0.92–1.04)). Patients had higher odds of receiving instrumental evaluations at hospitals in urban areas (urban = 7.2%; rural (ref) = 4.8%; OR (95% CI) = 1.83 (1.54–2.17)) and lower odds at small hospitals (< 200 bed = 5.5%; OR (95% CI) = 0.65 (0.53–0.79)) and medium-size hospitals (200–399 bed = 6.3%; OR (95% CI) = 0.75 (0.63–0.91)) compared to larger hospitals (≥ 400 bed (ref) = 8.5%). Utilization was also higher among males (males = 8.2%; females (ref) = 5.5%; OR (95% CI) = 1.46 (1.42–1.50)) and lower among Black (Black = 5.7%; White (ref) = 7.1%; OR (95% CI) = 0.82 (0.76–0.88)) and Hispanic individuals (Hispanic = 6.9%; non-Hispanic (ref) = 5.4%; OR (95% CI) = 0.87 (0.77–0.96)). See Table 2 for key significant model results and Table S7 for full model results.

### 3.4 | Dysphagia Therapy

Of all pneumonia patients in the study sample, 11.2% received dysphagia therapy. Higher utilization of dysphagia therapy was associated with ND diagnosis (ND = 24.1%; non-ND (ref) = 8.1%) and Asp-PNA diagnosis (Asp-PNA = 34.9%; CAP (ref) = 6.8%). The interaction between Asp-PNA and ND diagnosis was significant (OR (95% CI) = 0.48 (0.45–0.52)), indicating that while Asp-PNA was associated with 8.66 times greater odds of receiving dysphagia therapy in the non-ND group (OR (95% CI) = 8.66 (8.62–8.70)), the association was halved in the ND group (OR (95% CI) = 4.20 (3.98–4.43)). Similarly, having an ND diagnosis was associated with 3.11 times greater odds of receiving a clinical evaluation for those with CAP (OR (95% CI) = 3.11 (3.06–3.15)); however, the association between ND diagnosis and receiving a dysphagia therapy was dampened among those with Asp-PNA (OR (95% CI) = 1.50 (1.43–1.59)). Patients had higher odds of receiving dysphagia therapy at hospitals in urban areas (urban = 11.9%, rural = (ref) 7.6%; OR (95% CI) = 2.25 (1.91–2.66)). Therapy use was also higher for males (males = 12.3%; females = (ref) 10.2%; OR (95% CI) = 1.18 (1.15–1.22)). See Table 2 for key significant model results and Table S8 for full model results.

## 4 | Discussion

To our knowledge, this is the first study to examine dysphagia service utilization in a large national cohort of hospitalized older adults with pneumonia. In this sample of 195,782 older adults with pneumonia across 943 U.S. hospitals, we identified overall low rates of dysphagia service provision, with clinical evaluations utilized most frequently (23.6%) and instrumental evaluations utilized least frequently (6.8%). We examined patient- and hospital-level factors that were associated with receiving dysphagia services and identified similar trends of utilization across all three types of dysphagia services. The strongest association with dysphagia service utilization was a diagnosis of Asp-PNA, followed by a neurodegenerative diagnosis. Patients treated at hospitals in urban areas and at larger hospitals also received more dysphagia services.

It is not surprising that a diagnosis of Asp-PNA had the largest association with receiving dysphagia services, given that an Asp-PNA diagnosis is usually made when patients present with overt oropharyngeal aspiration symptoms or known risk factors [11, 31]—which one would expect to trigger a dysphagia consult. It is also possible that findings from the dysphagia consult resulted in an Asp-PNA diagnosis for some patients. Given that a diagnosis of Asp-PNA is, by definition, linked to the presence or suspicion of dysphagia, we might expect that all Asp-PNA patients should receive a dysphagia evaluation. While our findings confirmed our hypothesis that dysphagia service provision would be higher among patients with Asp-PNA (63.6%) consistent with findings from prior retrospective, single-site, smaller studies [5, 20, 21], they also highlight that a significant portion (36.4%) of patients with Asp-PNA do not receive any SLP services.

Moreover, the differential diagnosis between Asp-PNA and CAP is ambiguous, and these codes are often used interchangeably. There are no gold-standard diagnostic criteria to distinguish Asp-PNA from CAP; their clinical presentation is often identical, and chest imaging cannot reliably distinguish between them [12, 13, 32]. In fact, prior studies have shown that dysphagia is also a prevalent clinical finding among patients hospitalized with CAP [17, 18, 32–34] and a risk factor for the development of CAP among older adults [35]. Studies have identified clinical signs of dysphagia in 30%–50% of CAP patients [18, 33], and one study identified silent aspiration during sleep in 71% of patients with CAP compared to 10% of controls [36]. In one cohort of older adults hospitalized with CAP, 91.7% exhibited oropharyngeal dysphagia on videofluoroscopic evaluation [16]. Despite the evidence to suggest a high prevalence of dysphagia among older adults with CAP, in our sample, only 16.2% of patients with a general CAP diagnosis received a clinical dysphagia evaluation. Our finding is similar to findings from one previous study, which found that 16% of patients hospitalized with CAP received SLP services, the type of which were unspecified [21]. Moreover, in our sample, less than 4% of patients with CAP received an instrumental evaluation, and less than 7% received therapy. The low rates of dysphagia service provision to patients with CAP identified in the present study are concerning, given aspiration is a precursor for bacterial pneumonia pathogenesis in the majority of cases and Asp-PNA is likely under diagnosed [12]. CAP patients with impaired swallow safety experience greater pneumonia severity, worse healthcare outcomes, and higher in-hospital, 30-day, and one-year mortality [16, 17]. Additionally, oropharyngeal dysphagia has independently predicted re-hospitalization for patients with both aspiration- and non-aspiration pneumonia [18].

The observation that an Asp-PNA diagnosis was associated with a ninefold higher odds of receiving dysphagia services among those without a neurodegenerative disease indicates that provider diagnostic classification of pneumonia as either CAP or Asp-PNA may have a direct impact on whether or not dysphagia services are provided. This is critical because receiving dysphagia services may have long-term benefits for patient quality of life, health, and survival. Studies have shown that when dysphagia rehabilitation was initiated in patients with Asp-PNA, and particularly when it was initiated early, patients experienced shorter hospital stays, greater likelihood and faster return to an oral diet, and higher odds of being discharged on a fully oral diet [22, 23]. Receiving SLP services has been shown to lower mortality rates and increase the likelihood of discharge to a rehabilitation setting for Asp-PNA patients [20]. Future research aimed at understanding physicians’ diagnostic assignment and discriminatory process for CAP versus Asp-PNA is necessary to identify potential interventions aimed at improving identification and care pathways for individuals at high risk for dysphagia-associated pneumonia.

When patients were diagnosed with CAP, being in the ND group was associated with double the odds of receiving a clinical evaluation and triple the odds of receiving therapy, although this association was dampened when patients were diagnosed with aspiration pneumonia. We would expect that an ND diagnosis would catalyze a referral for dysphagia services, given that dysphagia is a ubiquitous sequela of most neurodegenerative diseases [37–39]. Neurodegenerative populations also have an increased risk for pneumonia-associated mortality, with Asp-PNA consistently reported as the leading cause of death across neurodegenerative diseases [7–9, 40]. Unsurprisingly, Asp-PNA was also more prevalent in the ND group. Despite the known risks, dysphagia evaluation was conducted in less than half of the ND group. The infrequent use of instrumental evaluation among the ND group (12.1%) is also concerning because silent aspiration (impaired cough reflex to aspiration) is highly prevalent [41]. Clinical evaluation alone is often insufficient to detect the presence and severity of dysphagia given that it does not allow for visualization of the swallow. While clinical evaluations are necessary to determine the immediate safety of oral intake, identify risk factors for aspiration, and develop hypotheses about swallowing physiology, they rely on visible signs and symptoms and do not provide adequate predictive value for determining the presence of aspiration, which needs to be verified through instrumental assessment [42, 43]. The infrequent use of instrumental evaluation provides evidence to support further inquiry into system- and clinician-level barriers to providing instrumental evaluation as well as the need to consider ways to increase the provision of these important diagnostic tests.

We also identified that patients in urban areas had significantly higher odds of receiving all types of dysphagia services than those in rural areas. This is likely, at least in part, due to the shortage of healthcare providers in rural areas [44, 45], and has been documented in other populations [46, 47]. While limited research has examined disparities in the provision of SLP services, one study identified that 64% of healthcare facilities in Alabama did not have an SLP on staff and reported that difficulty hiring and retaining medical SLPs was a barrier to service provision [44]. In contrast, a recent study found similar rates of SLP utilization in urban and rural areas of New York State [48]. To our knowledge, the present study is the first to identify a significant disparity in dysphagia service provision to older adults hospitalized with pneumonia—with fewer services provided in rural hospitals. More work is needed to understand and address this disparity in order to provide equitable dysphagia services across the country. While there was no difference in dysphagia service provision between academic and community hospitals (in contrast to our hypothesis), we did identify lower service provision at smaller hospitals (< 200 beds), which is in accordance with previous findings [48], and again indicates resource and staffing as potential barriers to dysphagia service provision. More research is needed to understand and address geographic and health system factors that affect dysphagia care delivery. These disparities may be modified through innovation and system-level changes (e.g., implementing telehealth, mobile imaging services, developing hospital protocols, introducing dysphagia screening led by nurses) to improve health outcomes related to pneumonia and dysphagia.

While the Premier Healthcare Database contains a large number of U.S. hospital admissions, there are limitations to using administrative claims data. It is possible that participating hospitals differ systematically from hospitals which are not included in the database. The database also does not include some types of potentially relevant clinical information, including type of dysphagia therapy provided, diet level/modifications, and non-billed dysphagia screening by nurses. Additionally, we dichotomized dysphagia service utilization across three main service types (clinical evaluation, instrumental evaluation, and therapy) as present or absent and did not consider the number and type of evaluations and/or therapy sessions provided. Moreover, directionality of findings cannot be determined from cross-sectional data and therefore the associations identified may be bidirectional, where pneumonia type influences dysphagia service provision and vice versa—understanding this dynamic relationship is an important area for future study. Finally, our analysis only examined service utilization during the index hospital admission. The amount and type of dysphagia services provided prior to and after admission are important areas for future study.

### 4.1 | Conclusions and Implications

This study provides detailed data on the utilization of dysphagia services in a large national cohort of older adults hospitalized with pneumonia. Our findings demonstrate an overall low rate of dysphagia service provision, despite its known role in the pathogenesis of pneumonia, association with worse health outcomes in this population, and calls for universal screening [16, 31]. The diagnostic ambiguity between CAP and Asp-PNA, inconsistent code assignment, and variability in dysphagia referral patterns within and across pneumonia diagnoses indicate a clear need for standardization in dysphagia service provision to patients hospitalized with all types of community onset pneumonia, whether they are diagnosed generally as CAP or specifically as Asp-PNA. We identified that a diagnosis of Asp-PNA was the factor most strongly associated with receiving dysphagia services. Provider- and system-level changes in diagnostic assignment (e.g., an increase in Asp-PNA coding) may substantially impact the likelihood of pneumonia patients receiving a dysphagia evaluation. However, even among those with an Asp-PNA diagnosis, 40% did not receive SLP services. Future, prospective studies are also needed to understand the directionality of the relationship between an Asp-PNA diagnosis and dysphagia consults—how often do physicians refer pneumonia patients to SLP services because they have an Asp-PNA diagnosis and how often does the SLP consult trigger the Asp-PNA diagnosis? It is critical to understand provider decision-making and facility-specific processes that influence both pneumonia coding and SLP service delivery pathways. Qualitative and mixed-methods research that engages key stakeholders (e.g., hospitalist physicians, SLPs, administrators) is critical to shed light on the beliefs, decision-making processes, and structures that underlie current practice patterns and guide next steps. Future work examining the impact of dysphagia evaluation and therapy on health outcomes is also necessary to inform the development of policies and protocols to enhance dysphagia service provision for older adults hospitalized with pneumonia.

## Supplementary Material

Supporting Information

Supporting Information

Additional supporting information can be found online in the Supporting Information section. Table S1: ICD-10 diagnosis codes for aspiration pneumonia (Asp-PNA) and community acquired pneumonia (CAP). Table S2: List of antibiotic treatment during emergency department visit or first day of admission. Table S3: Administrative billing codes for dysphagia services. Table S4: ICD-10 Codes used to identify the neurodegenerative disease (ND) cohort. Table S5: Demographics by neurodegenerative disease status. Table S6: Variables associated with utilization of clinical swallowing evaluations: full model results. Table S7: Variables associated with utilization of instrumental swallowing evaluations: full model results. Table S8: Variables associated with utilization of dysphagia therapy: full model results.

## Figures and Tables

**TABLE 1 |:** Demographics.

	All (*n* = 195,782)	Asp-PNA (*n* = 30,816; 15.7%)	CAP (*n* = 164,966; 84.3%)	ASD

Patient characteristics				
Age (SD) [range]	76.5 (8.96) [60–89]	78.5 (9.0) [60–89]	76.1 (8.91) [60–89]	26.8%
Sex, *n* (%)				17.3%
Female	101,219 (51.7%)	13,682 (44.4%)	87,597 (53.1%)	
Male	94,563 (48.3%)	17,134 (55.6%)	77,369 (46.9%)	
Race, *n* (%)				7.9%
Asian	4699 (2.4%)	1079 (3.5%)	3629 (2.2%)	0.6%
Black	21,144 (10.8%)	3266 (10.6%)	17,816 (10.8%)	2.9%
White	156,626 (80.0%)	24,314 (78.9%)	131,973 (80.0%)	0.2%
Other	13,509 (6.9%)	2126 (6.9%)	11,383 (6.9%)	
Ethnicity, *n* (%)				
Non-Hispanic	184,622 (94.3%)	29,183 (94.7%)	155,398 (94.2%)	2.5%
Hispanic	11,160 (5.7%)	1633 (5.3%)	9568 (5.8%)	
Insurance, *n* (%)				5.1%
Medicaid	9593 (4.9%)	1233 (4.0%)	8413 (5.1%)	11.1%
Medicare	164,261 (83.9%)	26,902 (87.3%)	137,417 (83.3%)	10.0%
Private	14,096 (7.2%)	1572 (5.1%)	12,372 (7.5%)	2.2%
Other	7831 (4.0%)	1109 (3.6%)	6764 (4.1%)	
Diagnosis, *n* (%)				
ND	37,808 (19.3%)	11,880 (38.6%)	25,928 (15.7%)	53.1%
Non-ND	157,974 (80.7%)	18,936 (61.4%)	139,038 (84.3%)	
Hospital characteristics				
Hospital size				7.7%
< 200 beds	67,936 (34.7%)	7858 (25.5%)	47,675 (28.9%)	1.3%
200–399 beds	55,602 (28.4%)	11,556 (37.5%)	60,707 (36.8%)	5.8%
≥ 400 beds	72,244 (36.9%)	11,402 (37.0%)	56,583 (34.3%)	
Hospital Type				8.0%
Academic	84,186 (43.0%)	14,268 (46.3%)	69,781 (42.3%)	
Community	111,596 (57.0%)	16,548 (53.7%)	95,185 (57.7%)	
Hospital rurality				12.2%
Urban	163,674 (83.6%)	26,902 (87.3%)	136,757 (82.9%)	
Rural	32,108 (16.4%)	3914 (12.7%)	28,209 (17.1%)	

Abbreviations: ASD = absolute standard difference; Asp-PNA = aspiration pneumonia; CAP = community acquired pneumonia; ND = neurodegenerative disease; non-ND = non-neurodegenerative disease.

**TABLE 2 |:** Key variables associated with utilization of clinical swallowing evaluations, instrumental swallowing evaluations, and dysphagia therapy.

Dysphagia service	Variables impacting dysphagia service utilization		

	ND	Non-ND^a^	OR (95% CI)
Clinical Eval, % (*n*)	45.8% (17,302)	18.3% (28,915)	2.85 (2.76–2.95)
Instrumental, % (*n*)	12.1% (4559)	5.6% (8770)	2.03 (1.91–2.16)
Therapy, % (*n*)	24.1% (9096)	8.1% (12,869)	3.11 (2.97–3.24)
	Asp-PNA	CAP^a^	OR (95% CI)
Clinical Eval, % (*n*)	63.6% (19,601)	16.1% (26,616)	9.57 (9.24–9.91)
Instrumental, % (*n*)	23.2% (7149)	3.7% (6180)	9.67 (9.21–10.14)
Therapy, % (*n*)	34.9% (10,743)	6.8% (11,222)	8.66 (8.31–9.03)
	Urban	Rural^a^	OR (95% CI)
Clinical Eval, % (*n*)	24.7% (40,401)	18.1% (5816)	1.57 (1.41–1.75)
Instrumental, % (*n*)	7.2% (11,786)	4.8% (1543)	1.83 (1.54–2.17)
Therapy, % (*n*)	11.9% (19, 536)	7.6% (2429)	2.25 (1.91–2.66)
	< 200 bed	≥ 400 bed^a,b^	OR (95% CI)
Clinical Eval, % (*n*)	21.1% (11,720)	25.5% (17,338)	0.84 (0.74–0.96)
Instrumental, % (*n*)	5.5% (3045)	8.5% (5778)	0.65 (0.53–0.79)
Therapy, % (*n*)	10.4% (5767)	11.6% (7879)	0.94 (0.77–1.14)

Abbreviations: Asp-PNA = aspiration pneumonia; CAP = community acquired pneumonia; CI = confidence interval; ND = neurodegenerative disease; non-ND = non-neurodegenerative disease; OR = odds ratio.

a Reference category.

b The middle category (200–399 beds) was excluded from this table because it was not significantly different from the other categories but is included in the full model results in Tables S4–S6.

## Summarys

- Key points
  - 75% of high-risk older adults with pneumonia admitted to U.S. hospitals did not receive any dysphagia services.
  - Across all dysphagia services (clinical evaluation, instrumental evaluation, and therapy) utilization was higher for individuals with Aspiration Pneumonia, individuals with neurodegenerative disease, and in urban hospitals; and lower in smaller (< 200 bed) hospitals.
  - A diagnosis of Aspiration Pneumonia was the strongest determinant of receiving dysphagia services; suggesting that provider- and system-level changes in diagnostic assignment (e.g., an increase in Asp-PNA coding) may substantially impact the likelihood of pneumonia patients receiving a dysphagia evaluation.
- Why does this paper matter?
  - Dysphagia plays a known role in the pathogenesis of pneumonia and association with worse health outcomes. Therefore, identifying factors that impact dysphagia service provision is necessary to enhance service provision and improve health outcomes for older adults hospitalized with pneumonia.
