# Supplementary material for: Utilization of Dysphagia Services Among Older Adults Hospitalized With Pneumonia in a Large Sample of US Hospitals
Source: J Am Geriatr Soc. 2025 Sep 27;73(11):3484–94. doi: 10.1111/jgs.70119 (PMC12581205; doi:10.1111/jgs.70119)
Supplement: Supplementary file 1 — Table S1: ICD‐10 diagnosis codes for aspiration pneumonia (Asp‐PNA) and community acquired pneumonia (CAP). Table S2: List of antibiotic treatment during emergency department visit or first day of admission. Table S3: Administrative billing codes for dysphagia services. Table S4: ICD‐10 Codes used to identify the neurodegenerative disease (ND) cohort. Table S5: Demographics by neurodegenerative disease status. Table S6: Variables associated with utilization of clinical swallowing evaluations: full model results. Table S7: Variables associated with utilization of instrumental swallowing evaluations: full model results. Table S8: Variables associated with utilization of dysphagia therapy: full model results. [file JGS-73-3484-s001.pdf]

**Supplemental Table 1. ICD-10 Diagnosis Codes for Aspiration Pneumonia (Asp-PNA) and Community Acquired Pneumonia (CAP)**

| Code | Description |
|---|---|
| **Aspiration Pneumonia (Asp-PNA)** | |
| J69.0 | Pneumonitis due to inhalation of food and vomit |
| J69.8 | Pneumonitis due to inhalation of other solids and liquids |
| **Community Acquired Pneumonia (CAP)** | |
| J13 | Pneumonia due to Streptococcus pneumoniae |
| J14 | Pneumonia due to Hemophilus influenzae |
| J15.0 | Pneumonia due to Klebsiella pneumoniae |
| J15.1 | Pneumonia due to Pseudomonas |
| J15.20 | Pneumonia due to staphylococcus, unspecified |
| J15.211 | Pneumonia due to Methicillin susceptible Staphylococcus aureus |
| J15.212 | Pneumonia due to Methicillin resistant Staphylococcus aureus |
| J15.29 | Pneumonia due to other staphylococcus |
| J15.3 | Pneumonia due to streptococcus, group B |
| J15.4 | Pneumonia due to other streptococci |
| J15.5 | Pneumonia due to Escherichia coli |
| J15.6 | Pneumonia due to other Gram-negative bacteria |
| J15.7 | Pneumonia due to Mycoplasma pneumoniae |
| J15.8 | Pneumonia due to other specified bacteria |
| J15.9 | Unspecified bacterial pneumonia |
| J16.0 | Chlamydial pneumonia |
| J16.8 | Pneumonia due to other specified infectious organisms |
| J17 | Pneumonia in diseases classified elsewhere |
| J18.0 | Bronchopneumonia, unspecified organism |
| J18.1 | Lobar pneumonia, unspecified organism |
| J18.8 | Other pneumonia, unspecified organism |
| J18.9 | Pneumonia, unspecified organism |
| J85.1 | Abscess of lung with pneumonia |

**Supplemental Table 2. List of Antibiotic Treatment during Emergency Department Visit or First Day of Admission**

| Antibiotic Name |
|---|
| 1. Cefotaxime |
| 2. Ceftriaxone |
| 3. Azithromycin |
| 4. Clarithromycin |
| 5. Ampicillin/Sulbac |
| 6. Levofloxacin |
| 7. Moxifloxacin |
| 8. Doxycycline |
| 9. Cefpodoxime |
| 10. Cefuroxime |
| 11. Amoxicillin |
| 12. Amox/Pot Clav |

**Supplemental Table 3. Administrative Billing Codes for Dysphagia Services**

| Premier Healthcare Billing Code | Associated CPT Code |
|---|---|
| **Clinical Swallowing Evaluation** | 92610 |
| ST EVAL SWALLOW ORAL & PHARYNGEAL FUNCTION | |
| ST EVAL SWALLOW 15 MIN | |
| ST EVAL SWALLOW 60 MIN | |
| ST EVAL ORAL FUNCTION | |
| ST EVAL SWALLOW 30 MIN | |
| PF ST EVAL SWALLOW | |
| **Instrumental Evaluation** | |
| ***Videofluoroscopic Evaluation of Swallowing (VFSS)*** | 92611 |
| ST EVAL SWALLOW FLUORO CINE/VIDEO | |
| ST EVAL SWALLOW VIDEO/FLUORO | |
| ST EVAL SWALLOW VIDEO/FLUORO 15 MIN | |
| XR PHARYNX/ESOPHAGUS SWALLOW CINE/VIDEO | |
| PF ST EVAL SWALLOW FLUORO CINE/VIDEO | |
| ***Flexible Endoscopic Evaluation of Swallowing (FEES)*** | |
| ST EVAL SWALLOW ENDO CINE/VIDEO | 92612 |
| ST EVAL ENDO SWALLOW/SENSORY TEST CINE/VIDEO | 92616 |
| **Therapy** | 92526 |
| ST TREATMENT SWALLOW | |
| ST TREATMENT SWALLOW 15 MIN | |
| PF ST TREATMENT SWALLOW 15 MIN | |

**Supplemental Table 4. ICD-10 Codes Used to Identify the Neurodegenerative Disease (ND) Cohort**

| Neurodegenerative Disease Category | ICD-10 Code | ICD-10 Diagnosis |
|---|---|---|
| **Alzheimer's disease** | G30.0 | Alzheimer's disease with early onset |
| | G30.1 | Alzheimer's disease with late onset |
| | G30.8 | Other Alzheimer's disease |
| | G30.9 | Alzheimer's disease, unspecified |
| **Vascular Dementia** | F01.50 | Vascular dementia without behavioral disturbance |
| | F01.51 | Vascular dementia with behavioral disturbance |
| **Dementia in other diseases** | F02.80 | Dementia in other diseases classified elsewhere, unspecified severity, without behavioral disturbance, psychotic disturbance, mood disturbance, and anxiety |
| | F02.81 | Dementia in other diseases classified elsewhere with behavioral disturbance |
| | F02.811 | Dementia in other diseases classified elsewhere, unspecified severity, with agitation |
| | F02.818 | Dementia in other diseases classified elsewhere, unspecified severity, with other behavioral disturbance |
| | F02.82 | Dementia in other diseases classified elsewhere, unspecified severity, with psychotic disturbance |
| | F02.83 | Dementia in other diseases classified elsewhere, unspecified severity, with mood disturbance |
| | F02.84 | Dementia in other diseases classified elsewhere, unspecified severity, with anxiety |
| | F02.A0 | Dementia in other diseases classified elsewhere, mild, without behavioral disturbance, psychotic disturbance, mood disturbance, and anxiety |
| | F02.A11 | Dementia in other diseases classified elsewhere, mild, with agitation |
| | F02.A18 | Dementia in other diseases classified elsewhere, mild, with other behavioral disturbance |
| | F02.A2 | Dementia in other diseases classified elsewhere, mild, with psychotic disturbance |
| | F02.A3 | Dementia in other diseases classified elsewhere, mild, with mood disturbance |
| | F02.A4 | Dementia in other diseases classified elsewhere, mild, with anxiety |

| | | |
|---|---|---|
| | F02.B0 | Dementia in other diseases classified elsewhere, moderate, without behavioral disturbance, psychotic disturbance, mood disturbance, and anxiety |
| | F02.B11 | Dementia in other diseases classified elsewhere, moderate, with agitation |
| | F02.B18 | Dementia in other diseases classified elsewhere, moderate, with other behavioral disturbance |
| | F02.B2 | Dementia in other diseases classified elsewhere, moderate, with psychotic disturbance |
| | F02.B3 | Dementia in other diseases classified elsewhere, moderate, with mood disturbance |
| | F02.B4 | Dementia in other diseases classified elsewhere, moderate, with anxiety |
| | F02.C0 | Dementia in other diseases classified elsewhere, severe, without behavioral disturbance, psychotic disturbance, mood disturbance, and anxiety |
| | F02.C11 | Dementia in other diseases classified elsewhere, severe, with agitation |
| | F02.C18 | Dementia in other diseases classified elsewhere, severe, with other behavioral disturbance |
| | F02.C2 | Dementia in other diseases classified elsewhere, severe, with psychotic disturbance |
| | F02.C3 | Dementia in other diseases classified elsewhere, severe, with mood disturbance |
| | F02.C4 | Dementia in other diseases classified elsewhere, severe, with anxiety |
| | G31.84 | Mild Cognitive impairment, so stated |
| **Unspecified Dementia** | F03.90 | Unspecified dementia, unspecified severity, without behavioral disturbance, psychotic disturbance, mood disturbance, and anxiety |
| | F03.91 | Unspecified dementia with behavioral disturbance |
| | F03.911 | Unspecified dementia, unspecified severity, with agitation |
| | F03.918 | Unspecified dementia, unspecified severity, with other behavioral disturbance |
| | F03.92 | Unspecified dementia, unspecified severity, with psychotic disturbance |
| | F03.93 | Unspecified dementia, unspecified severity, with mood disturbance |
| | F03.94 | Unspecified dementia, unspecified severity, with anxiety |
| | F03.A0 | Unspecified dementia, mild, without behavioral disturbance, psychotic disturbance, mood disturbance, and anxiety |
| | F03.A11 | Unspecified dementia, mild, with agitation |
| | F03.A18 | Unspecified dementia, mild, with other behavioral disturbance |
| | F03.A2 | Unspecified dementia, mild, with psychotic disturbance |
| | F03.A3 | Unspecified dementia, mild, with mood disturbance |

| | | |
|---|---|---|
| | F03.A4 | Unspecified dementia, mild, with anxiety |
| | F03.B0 | Unspecified dementia, moderate, without behavioral disturbance, psychotic disturbance, mood disturbance, and anxiety |
| | F03.B11 | Unspecified dementia, moderate, with agitation |
| | F03.B18 | Unspecified dementia, moderate, with other behavioral disturbance |
| | F03.B2 | Unspecified dementia, moderate, with psychotic disturbance |
| | F03.B3 | Unspecified dementia, moderate, with mood disturbance |
| | F03.B4 | Unspecified dementia, moderate, with anxiety |
| | F03.C0 | Unspecified dementia, severe, without behavioral disturbance, psychotic disturbance, mood disturbance, and anxiety |
| | F03.C11 | Unspecified dementia, severe, with agitation |
| | F03.C18 | Unspecified dementia, severe, with other behavioral disturbance |
| | F03.C2 | Unspecified dementia, severe, with psychotic disturbance |
| | F03.C3 | Unspecified dementia, severe, with mood disturbance |
| | F03.C4 | Unspecified dementia, severe, with anxiety |
| **Frontotemporal dementia** | G31.09 | Other frontotemporal dementia |
| | G31.01 | Pick’s disease |
| **Parkinson’s Disease and Parkinsonism** | G20 | Parkinson's disease |
| | G23.1 | Progressive supranuclear palsy |
| | G23.2 | Striatonigral degeneration |
| | G23.8 | Other specified disease of the basal ganglia |
| | G23.9 | Degenerative disease of basal ganglia, unspecified |
| | G90.3 | Multiple system atrophy |
| | G31.83 | Dementia with Lewy bodies |
| | G31.85 | Corticobasal Degeneration |
| **Secondary Parkinsonism** | G21.0 | Malignant neuroleptic syndrome |
| | G21.1 | Other drug-induced secondary parkinsonism |
| | G21.2 | Secondary parkinsonism due to other external agents |
| | G21.3 | Postencephalitic parkinsonism |
| | G21.4 | Vascular parkinsonism |
| | G21.8 | Other secondary parkinsonism |
| | G21.9 | Secondary parkinsonism, unspecified |
| **Amyotrophic Lateral Sclerosis and other Neuromuscular Disorders** | G12.21 | Amyotrophic lateral sclerosis (ALS) |
| | G12.0 | Infantile spinal muscular atrophy, type I [Werdnig-Hoffman] |
| | G12.1 | Other inherited spinal muscular atrophy |
| | G12.8 | Other spinal muscular atrophies and related syndromes |
| | G12.9 | Spinal muscular atrophy, unspecified |
| | G12.20 | Motor neuron disease, unspecified |

| | | |
|---|---|---|
| | G12.22 | Primary lateral sclerosis |
| | G12.23 | Progressive bulbar palsy |
| | G12.24 | Pseudobulbar palsy |
| **Multiple Sclerosis** | G35 | Multiple sclerosis |
| | G36.0 | Neuromyelitis optica (Devic) |
| | G73.3 | Acute transverse myelitis in demyelinating disease of the CNS |
| | G37.9 | Demyelinating disease of the CNS, unspecified |
| **Huntington's Disease** | G10 | Huntington's disease (also known as Huntington's chorea) |
| **Cerebellar Ataxia** | **G11.1** | Early-onset cerebellar ataxia (includes Freidrich's ataxia) |
| | **G11.2** | Late-onset cerebellar ataxia |
| | **G11.3** | Cerebellar ataxia with defective DNA repair |
| | **G11.4** | Hereditary spastic paraplegia (SCA) |
| | **G11.8** | Other hereditary ataxias |
| | **G11.9** | Hereditary ataxia, unspecified |
| | R26.0 | Ataxic gait |
| | R27.0 | Ataxia, unspecified |
| **Wilson's disease** | E83.0 | Wilson's disease |
| **Myasthenia Gravis** | G70.00 | Myasthenia Gravis |
| | G70.01 | Myasthenia gravis with (acute) exacerbation |
| | G70.1 | Toxic Myoneural disorders |
| | G70.2 | Congenital and developmental myasthenia |
| | G70.80 | Lambert-Eaton syndrome, unspecified |
| | G70.81 | Lambert-Eaton syndrome in disease classified elsewhere |
| | G70.89 | Other specified myoneural disorders |
| | G70.9 | Myoneural disorder, unspecified |
| **Guillain Barre Syndrome** | G61.0 | Guillain-Barre syndrome |
| **Prion diseases** | A81.0 | Creutzfeldt-Jakob disease |
| | A81.00 | Creutzfeldt-Jakob disease, unspecified |
| | A81.01 | Variant Creutzfeldt-Jakob disease |
| | A81.09 | Other Creutzfeldt-Jakob disease |
| | A81.1 | Subacute sclerosing panencephalitis |
| | A81.2 | Progressive multifocal leukoencephalopathy |
| | A81.81 | Kuru |
| | A81.82 | Gerstmann-Sträussler-Scheinker syndrome |
| | A81.83 | Fatal familial insomnia |
| | A81.89 | Other atypical virus infections of central nervous system |

| | | |
|---|---|---|
| | A81.9 | Atypical virus infections of the central nervous system, unspecified (can include prion diseases not specified elsewhere) |
| **Other rare neurodegenerative diagnoses** | G23.0 | Hallervorden-Spatz disease (often used for the entire NBIA group) |
| | G60.0 | Hereditary neuropathy with liability to pressure palsies |
| | E75.24 | Niemann-Pick disease |
| | E75.240 | Niemann-Pick disease type A |
| | E75.241 | Niemann-Pick disease type B |
| | E75.242 | Niemann-Pick disease type C |
| | E75.243 | Niemann-Pick disease type D |
| | E75.244 | Niemann-Pick disease type A/B |
| | E75.248 | Other Niemann-Pick disease |
| | E75.249 | Niemann-Pick disease, unspecified |

ICD-10 = International Classification of Diseases, Tenth Revision.

**Supplemental Table 5. Demographics by Neurodegenerative Disease Status**

| | All (n =195,782) | Non-ND (n =157,974; 80.7%) | ND (n =37,808; 19.3%) | ASD |
|---|---|---|---|---|
| ***Patient Characteristics*** | | | | |
| Age (SD) [range] | 76.5 (8.96) [60 – 89] | 75.2 (8.86) [60 – 89] | 81.5 (7.53) [60 – 89] | 76% |
| Sex, n (%) | | | | |
| Female | 101,219 (51.7%) | 81,199 (51.4%) | 20,038 (53.0%) | 3.2% |
| Male | 94,563 (48.3%) | 76,775 (48.6%) | 17,770 (47.0%) | |
| Race, n (%) | | | | |
| Asian | 4,699 (2.4%) | 3,475 (2.2%) | 1,210 (3.2%) | 5.8% |
| Black | 21,144 (10.8%) | 17,219 (10.9%) | 3,894 (10.3%) | 2.0% |
| White | 156,626 (80.0%) | 126,537 (80.1%) | 29,944 (79.2%) | 2.3% |
| Other | 13,509 (6.9%) | 10,742 (6.8%) | 2,798 (7.4%) | 2.3% |
| Ethnicity, n (%) | | | | |
| Non-Hispanic | 184,622 (94.3%) | 14,8969 (94.3%) | 35,464 (93.8%) | 2.4% |
| Hispanic | 11,160 (5.7%) | 8847 (5.6%) | 2,344 (6.2%) | |
| Insurance, n (%) | | | | |
| Medicaid | 9,593 (4.9%) | 8,689 (5.5%) | 870 (2.3%) | 16.5% |
| Medicare | 164,261 (83.9%) | 129,855 (82.2%) | 34,557 (91.4%) | 27.6% |
| Private | 14,096 (7.2%) | 12,796 (8.1%) | 1,172 (3.1%) | 21.9% |
| Other | 7,831 (4.0%) | 6,635 (4.2%) | 1,172 (3.1%) | 5.6% |
| PNA Type, n (%) | | | | |
| CAP | 164,966 (84.3%) | 139,038 (88.0%) | 25,928 (68.6%) | 48.5% |
| Asp-PNA | 30,816 (15.7%) | 18,936 (12.0%) | 11,880 (31.4%) | |
| ***Hospital Characteristics*** | | | | |

| | | | | |
|---|---|---|---|---|
| Hospital Size | | | | |
| <200 beds | 67,936 (34.7%) | 45,181 (28.6%) | 10,435 (27.6%) | 2.4% |
| 200-399 beds | 55,602 (28.4%) | 58,450 (37.0%) | 13,913 (36.8%) | 0.4% |
| ≥400 beds | 72,244 (36.9%) | 54,501 (34.5%) | 13,460 (35.6%) | 2.2% |
| Hospital Type | | | | |
| Academic | 84,186 (43.0%) | 67,139 (42.5%) | 16,938 (44.8%) | 4.6% |
| Community | 111,596 (57.0%) | 90,835 (57.5%) | 20,870 (55.2%) | |
| Hospital Rurality | | | | |
| Urban | 163,674 (83.6%) | 131,592 (83.3%) | 32,099 (84.9%) | 4.3% |
| Rural | 32,108 (16.4%) | 26,382 (16.7%) | 5,709 (15.1%) | |

Asp-PNA = Aspiration Pneumonia; CAP = Community Acquired Pneumonia; ASD = Absolute Standard Difference; ND = neurodegenerative disease; non-ND = non-neurodegenerative disease.

**Supplemental Table 6. Variables Associated with Utilization of Clinical Swallowing Evaluations: Full Model Results**

| | Unadjusted Model Results | | Adjusted Model Results | |
|---|---|---|---|---|
| Variable | OR (95% CI) | p-value | OR (95% CI) | p-value |
| (Intercept) | | | 0.09 (0.07 – 0.10) | <.001 |
| Pneumonia Type | | | | |
| Asp-PNA | 9.08 (8.88-9.33) | <.001 | 9.57 (9.24 – 9.91) | <.001 |
| CAP | Ref | | Ref | |
| Diagnosis | | | | |
| ND | 3.77 (3.68 – 3.86) | <.001 | 2.85 (2.76 – 2.95) | <.001 |
| Non-ND | Ref | | Ref | |
| Pneumonia Type * Diagnosis | N/A | | 0.57 (0.54 – 0.61) | <.001 |
| Asp-PNA in ND | | | 5.49 (5.22 – 5.77) | |
| ND in Asp-PNA | | | 1.64 (1.55 – 1.72) | |
| Age, years | 1.06 (1.05 – 1.06) | <.001 | 1.04 (1.03 – 1.06) | <.001 |
| Gender | | | | |
| Male | 1.17 (1.15 – 1.20) | <.001 | 1.13 (1.11 – 1.16) | <.001 |
| Female | Ref | | Ref | |
| Race | | | | |
| Asian | 1.50 (1.41 – 1.5) | <.001 | 1.14 (1.05 – 1.24) | 0.002 |
| Black | 0.85 (0.82 – 0.88) | <.001 | 1.01 (0.96 – 1.05) | 0.710 |
| Other | 0.87 (0.84 – 0.91) | <.001 | 0.95 (0. 90 – 1.01) | 0.102 |
| White | Ref | | Ref | |
| Ethnicity | | | | |
| Hispanic | 0.91 (0.87 – 0.95) | <.001 | 0.94 (0.88 – 1.00) | 0.041 |
| Non-Hispanic | Ref | | Ref | |
| Rurality | | | | |
| Urban | 1.48 (1.43 – 1.52) | <.001 | 1.57 (1.41 – 1.75) | <.001 |
| Rural | Ref | | Ref | |
| Hospital Size | | | | |
| <200 bed | 0.78 (0.76 – 0.80) | <.001 | 0.84 (0.74 – 0.96) | 0.009 |
| 200-399 beds | 0.91 (0.88 – 0.93) | <.001 | 0.95 (0.84 – 1.07) | 0.422 |

| | | | | |
|---|---|---|---|---|
| ≥400 beds | Ref | | Ref | |
| Hospital type | | | | |
| Academic | 1.14 (1.12 – 1.17) | <.001 | 0.99 (0.90 – 1.10) | 0.927 |
| Community | Ref | | Ref | |
| | | | **Random Effects** | |
| | | | ICC = 0.087 | |
| | | | Random Effect Variance = 0.3144 | |

OR = Odds Ratio; CI = Confidence Interval; Asp-PNA = Aspiration Pneumonia; CAP = Community Acquired Pneumonia; ND = neurodegenerative disease, non-ND = non-neurodegenerative disease. ICC = Intraclass Correlation Coefficient

**Supplemental Table 7. Variables Associated with Utilization of Instrumental Swallowing Evaluations: Full Model Results**

| | Unadjusted Model Results | | Adjusted Model Results | |
|---|---|---|---|---|
| Variable | OR (95% CI) | p-value | OR (95% CI) | p-value |
| (Intercept) | | | 0.02 (0.01 - 0.02) | <.001 |
| Pneumonia Type | | | | |
| Asp-PNA | 7.76 (7.48 – 8.05) | <.001 | 9.67 (9.21 – 10.14) | <.001 |
| CAP | Ref | | Ref | |
| Diagnosis | | | | |
| ND | 2.33 (2.25 – 2.42) | <.001 | 2.03 (1.91- 2.16) | <.001 |
| Non-ND | Ref | | Ref | |
| Pneumonia Type * Diagnosis | N/A | | 0.48 (0.44 – 0.52) | <.001 |
| Asp-PNA in ND Group | | | 4.66 (4.35 – 4.99) | |
| ND in Asp-PNA Group | | | 0.98 (0.92 – 1.04) | |
| Age (years) | 1.04 (1.03 – 1.04) | <.001 | 1.03 (1.00 – 1.05) | <.001 |
| Gender | | | | |
| Male | 1.52 (1.47 – 1.58) | <.001 | 1.46 (1.40 – 1.52) | <.001 |
| Female | Ref | | Ref | |
| Race | | | | |
| Asian | 1.02 (0.92 – 1.15) | 0.680 | 0.97 (0.85 – 1.12) | 0.697 |
| Black | 0.80 (0.75 – 0.85) | <.001 | 0.82 (0.76 – 0.88) | <.001 |
| Other | 0.75 (0.69 – 0.81) | <.001 | 0.92 (0.834 – 1.01) | 0.079 |
| White | Ref | | Ref | |
| Ethnicity | | | | |
| Hispanic | 0.78 (0.71 – 0.84) | <.001 | 0.87 (0.78 – 0.96) | 0.008 |
| Non-Hispanic | Ref | | Ref | |
| Rurality | | | | |
| Urban | 1.54 (1.45 – 1.62) | <.001 | 1.83 (1.54 – 2.17) | <.001 |

| | | | | |
|---|---|---|---|---|
| Rural | Ref | | Ref | |
| Hospital Size | | | | |
| <200 bed | 0.62 (0.60 – 0.65) | <.001 | 0.65 (0.53 – 0.79) | <.001 |
| 200-399 bed | 0.71 (0.69 – 0.74) | <.001 | 0.75 (0.63 – 0.91) | 0.003 |
| ≥400 bed | | | Ref | |
| Hospital Type | | | | |
| Academic | 1.19 (1.15 – 1.24) | <.001 | 0.89 (0.76 – 1.04) | 0.151 |
| Community | Ref | | Ref | |
| | | | **Random Effects** | |
| | | | ICC = 0.177 | |
| | | | Random Effect Variance = 0.709 | |

OR = Odds Ratio; CI = Confidence Interval; Asp-PNA = Aspiration Pneumonia; CAP = Community Acquired Pneumonia; ND = neurodegenerative disease, non-ND = non-neurodegenerative disease. ICC = Intraclass Correlation Coefficient

**Supplemental Table 8. Variables Associated with Utilization of Dysphagia Therapy: Full Model Results**

| | Unadjusted Model Results | | Adjusted Model Results | |
|---|---|---|---|---|
| Variable | OR (95% CI) | p-value | OR (95% CI) | p-value |
| (Intercept) | | | 0.02 (0.01 – 0.02) | <.001 |
| Pneumonia Type | | | | |
| Asp-PNA | 7.33 (7.11 – 7.56) | <.001 | 8.66 (8.31 – 9.03) | <.001 |
| CAP | Ref | | | |
| Diagnosis | | | | |
| ND | 3.57 (3.47 – 3.68) | <.001 | 3.11 (2.97 – 3.24) | <.001 |
| Non-ND | Ref | | | |
| Pneumonia Type * Diagnosis | N/A | | 0.48 (0.45 – 0.52) | <.001 |
| Asp-PNA in ND Group | | | 4.20 (3.98 – 4.43) | |
| ND in Asp-PNA Group | | | 1.50 (1.43 – 1.59) | |
| Age (years) | 1.05 (1.05 – 1.05) | <.001 | 1.04 (1.02 – 1.05) | <.001 |
| Gender | | | | |
| Male | 1.24 (1.20 – 1.27) | <.001 | 1.18 (1.15 – 1.22) | <.001 |
| Female | Ref | | | |
| Race | | | | |
| Asian | 1.39 (1.28 – 1.51) | <.001 | 1.04 (0.94 – 1.16) | 0.440 |
| Black | 0.80 (0.76 – 0.84) | <.001 | 1.00 (0.95 – 1.06) | 0.913 |
| Other | 0.84 (0.79 – 0.89) | <.001 | 0.94 (0.87 – 1.01) | 0.103 |
| White | Ref | | | |
| Ethnicity | | | | |
| Hispanic | 0.95 (0.89 – 1.01) | 0.121 | 0.93 (0.86 – 1.01) | 0.069 |
| Non-Hispanic | Ref | | | |
| Rurality | | | | |
| Urban | 1.65 (1.58 – 1.73) | <.001 | 2.25 (1.91 – 2.66) | <.001 |
| Rural | Ref | | | |

| | | | | |
|---|---|---|---|---|
| Hospital Size | | | | |
| <200 bed | 0.88 (0.85 – 0.92) | <.001 | 0.94 (0.77 – 1.14) | 0.506 |
| 200-399 bed | 0.99 (0.96 – 1.02) | 0.476 | 1.11 (0.93 – 1.33) | 0.253 |
| ≥400 bed | Ref | | | |
| Hospital Type | | | | |
| Academic | 1.07 (1.04 – 1.11) | <.001 | 0.93 (0.80 – 1.08) | 0.335 |
| Community | Ref | | | |
| | | | **Random Effects** | |
| | | | ICC = 0.181 | |
| | | | Random Effect Variance = 0.728 | |

OR = Odds Ratio; CI = Confidence Interval; Asp-PNA = Aspiration Pneumonia; CAP = Community Acquired Pneumonia; ND = neurodegenerative disease, non-ND = non-neurodegenerative disease. ICC = Intraclass Correlation Coefficient
